# Calcium Electrochemotherapy for Tumor Eradication and the Potential of High-Frequency Nanosecond Protocols

**DOI:** 10.3390/ph16081083

**Published:** 2023-07-31

**Authors:** Eivina Radzevičiūtė-Valčiukė, Augustinas Želvys, Eglė Mickevičiūtė, Jovita Gečaitė, Auksė Zinkevičienė, Veronika Malyško-Ptašinskė, Vytautas Kašėta, Jurij Novickij, Tatjana Ivaškienė, Vitalij Novickij

**Affiliations:** 1 Department of Immunology, State Research Institute Centre for Innovative Medicine, 08406 Vilnius, Lithuania; augustinas.zelvys@imcentras.lt (A.Ž.); jovita.gecaite@gmc.stud.vu.lt (J.G.); aukse.zinkeviciene@imcentras.lt (A.Z.); tatjana.ivaskiene@imcentras.lt (T.I.); 2 Faculty of Electronics, Vilnius Gediminas Technical University, 08412 Vilnius, Lithuania; veronika.malysko-ptasinske@vilniustech.lt (V.M.-P.); jurij.novickij@vilniustech.lt (J.N.); 3 Department of Biomodels, State Research Institute Centre for Innovative Medicine, 11342 Vilnius, Lithuania; egle.mickeviciute@imcentras.lt (E.M.); vytautas.kaseta@imcentras.lt (V.K.)

**Keywords:** cancer, calcium, nanosecond, electroporation, immunology, immunomodulation, anticancer, in vivo

## Abstract

Calcium electroporation (CaEP) is an innovative approach to treating cancer, involving the internalization of supraphysiological amounts of calcium through electroporation, which leads to cell death. CaEP enables the replacement of chemotherapeutics (e.g., bleomycin). Here, we present a standard microsecond (μsCaEP) and novel high-frequency nanosecond protocols for calcium electroporation (nsCaEP) for the elimination of carcinoma tumors in C57BL/6J mice. We show the efficacy of CaEP in eliminating tumors and increasing their survival rates in vivo. The antitumor immune response after the treatment was observed by investigating immune cell populations in tumors, spleens, lymph nodes, and blood, as well as assessing antitumor antibodies. CaEP treatment resulted in an increased percentage of CD4^+^ and CD8^+^ central memory T cells and decreased splenic myeloid-derived suppressor cells (MDSC). Moreover, increased levels of antitumor IgG antibodies after CaEP treatment were detected. The experimental results demonstrated that the administration of CaEP led to tumor growth delay, increased survival rates, and stimulated immune response, indicating a potential synergistic relationship between CaEP and immunotherapy.

## 1. Introduction

Oncological diseases are one of the most common causes of death worldwide and cancer treatment has always been a challenging problem [1]. Traditional treatment methods such as surgery, chemotherapy, and radiotherapy have been widely utilized [2]. Drawbacks associated with conventional chemotherapy include challenges in determining appropriate dosage, lack of specificity, rapid drug metabolism, and the occurrence of harmful side effects [3]. So, there is a growing need to explore alternative and more potent anticancer treatment strategies. A promising approach involves electroporation (EP) combined with chemotherapeutics, calcium ions, or other pharmaceuticals, offering a novel therapeutic treatment modality [4].

EP can be described as a physical phenomenon when applied pulsed electric fields (PEFs) induce cell membrane permeabilization by aqueous pores formation, which allows for the passage of non-permeable ions or molecules into the cell [5,6,7]. EP can be performed using different pulse settings (amplitude, pulse duration, number of pulses, etc.) [8,9]. Depending on the pulsed power input, the formed pores in the cell membrane can be transient (reversible EP) or the cell plasma membrane can be disrupted permanently (irreversible EP) [7,10]. The methodology had shown clinical potential in cancer treatment as a tissue ablation method or when combined with initially non-permeant chemotherapeutics to increase their uptake (electrochemotherapy (ECT)) [5,6,11]. Recently, the potential of EP in combination with calcium ions has been highlighted (calcium electroporation (CaEP)), which established a new modality of localized ECT [12,13,14]. It was shown that cancer cells are more sensitive to CaEP when compared to healthy cells, presumably due to distinct membrane repair abilities, varying membrane compositions, diverse energy reserves, and other distinguishing characteristics [13,15,16].

Since 2012, clinical studies have typically involved standard EP operating procedure protocol using eight high voltage 100 μs monopolar electric pulses with a repetition frequency of 1 Hz (ESOPE). The methodology is safe with good treatment efficacy; however, pain and muscle contractions could be highlighted as side effects [17,18]. Additionally, ensuring a homogeneous treatment is challenging, especially with non-invasive electrodes and highly heterogeneous tissue structures [19]. To compensate, higher frequency pulses can be used for impedance mitigation and equivalent efficacy nanosecond pulse protocols can be derived, which was confirmed in vitro [20,21,22,23] and in vivo [19,24,25,26,27,28]. High-frequency short-duration protocols show potential advantages compared to standard EP treatment, such as minimized thermal damage [29], reduced neuromuscular stimulation [30], lower Joule heating [31], and most importantly, a more homogeneous response with high flexibility in parametric protocol design.

In vitro CaEP studies revealed that cancer cell killing efficacy is comparable with bleomycin or cisplatin [32,33,34,35,36], and the treatment-induced death depends on calcium concentration (0.4–5.0 mM) [6,12,34,35,37]. Calcium concentrations up to 5 mM without EP are non-toxic in vitro. However, for successful in vivo or clinical setting Ca^2+^ concentration is increased up to 500 mM, typically injecting a volume corresponding to half of the tumor [13]. Additionally, it has been reported that CaEP has an anti-vascular effect on tumors in vivo [36].

From the biophysical point of view, Ca^2+^ is an essential second messenger ion involved in numerous vital cellular processes from transcription, proliferation, and metabolism to cell death [5,10,12,38]. In order to survive, the cells regulate intracellular calcium concentration, while in the presence of cell membrane permeabilization intracellular calcium concentration increases, this leads to adenosine triphosphate (ATP) depletion [35,36], mitochondrial dysfunction, and cell death by necrosis [12,14,34,38,39]. Moreover, the presence of higher calcium levels could potentially lead to the production of reactive oxygen species (ROS) and trigger the activation of lipases and proteases [39,40]. It is well known that undergoing necrosis causes membrane rapture and intracellular content release, which can be a potential immune response stimulator [41]. In addition, it has been reported that CaEP in vitro induces the release of the High Mobility Group Box 1 protein (HMGB1), which is a damage-associated molecular pattern (DAMP) molecule important in the immunogenic cell death (ICD) [41,42]. The release of such molecules stimulates a systemic immune response, including specific immunity.

In vivo colon cancer study with immunocompetent mice showed that after CaEP treatment, the fully recovered mice did not have tumors after being rechallenged with the same cancer cell line. Moreover, calcium electroporation treatment increased the systemic release of pro-inflammatory cytokines [5]. Previously, we have shown that CaEP increases T cell numbers, decreases the number of suppressor cells, and elevates the level of tumor cell-specific antibodies in the mice sera [26]. It was also reported that CaEP prolongs the survival of animals and stimulates the innate immune system when combined with gene electrotransfer of plasmid encoding IL-12 [35]. Moreover, a clinical study with CaEP indicated a systemic immune response after the treatment. Patients with malignant melanoma had complete remission in both treated and untreated metastases, even without any additional treatment [43,44].

Recently, a new modality of EP was proposed, which is based on nanosecond pulses compressed into an MHz burst [45,46]. When the delay between the pulses is shorter than the polarization constant of biological cells, a residual transmembrane voltage is formed throughout the sequence [46]. As a result, the electrotransfer of drugs or genes is significantly improved, enabling derivation of equivalent efficiency or even superior to ESOPE protocols [21,47]. The high potential of MHz burst of pulses was confirmed in vivo using bleomycin electrochemotherapy [27,48].

This work is the first in vivo study characterizing the effects of the MHz pulses for CaEP and the corresponding changes in the immune system response.

## 2. Results

### 2.1. Tumor Reduction and Increased Survival by Different Modalities of Calcium Electroporation

**Figure 1:**
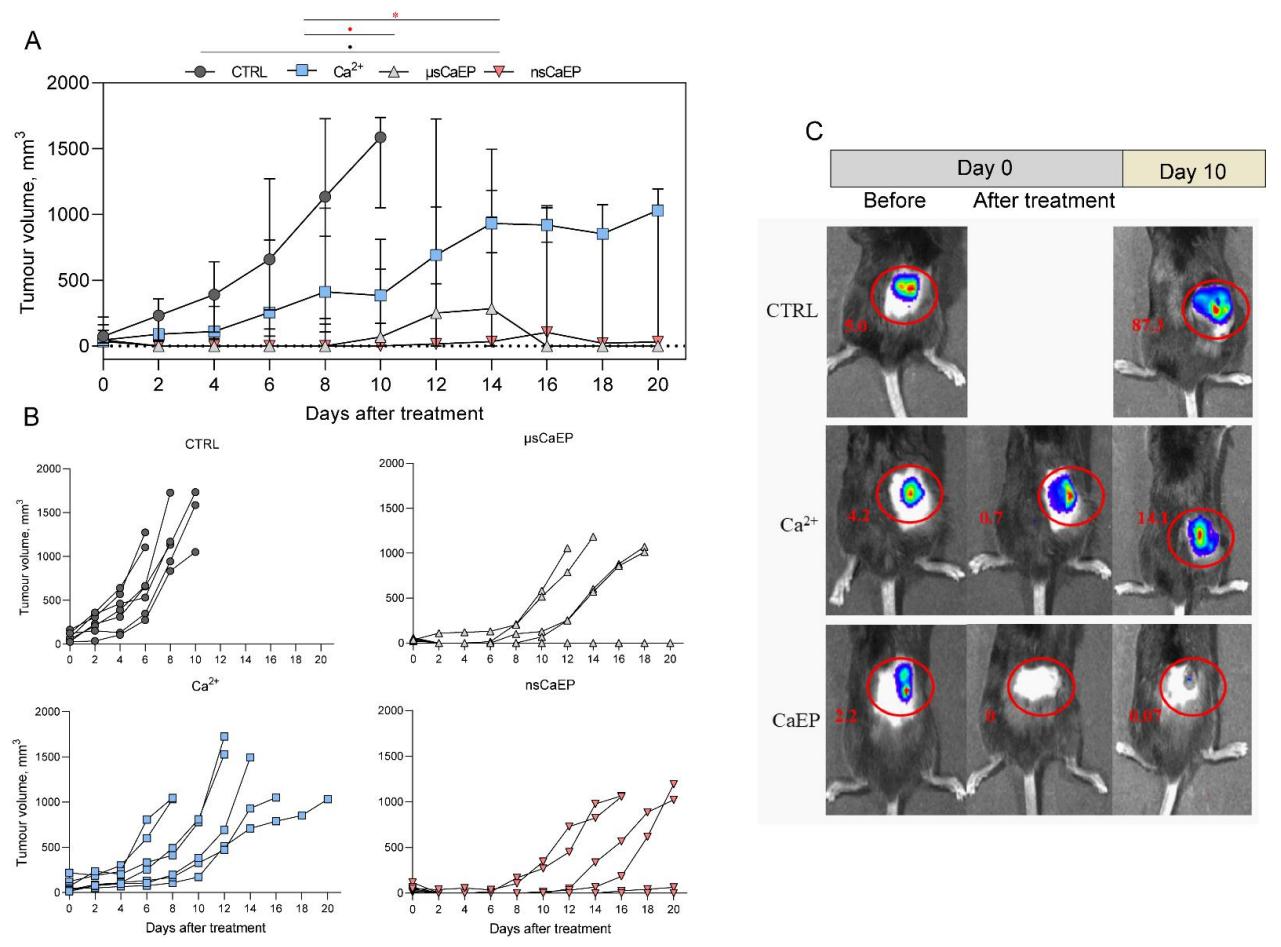
(**A**) Volumetric tumor growth changes after the treatment. (**B**) Volumetric tumor growth dynamics of separate animals in each treatment group. (**C**) Representative tumors luminescence images from an IVIS Spectrum device and Living Image software total fluxes (p/s × 10^6^) are marked in red, where CTRL—untreated tumor-bearing mice; and Ca^2+^—tumor-bearing mice treated with CaCl_2_ (in 0.9% NaCl solution); μsCaEP—1.5 kV/cm × 100 μs × 8, 1 Hz protocol; nsCaEP—4 kV/cm × 700 ns × 200, 1 MHz protocol. Symbols in black correspond to statistically significant (* *p* < 0.05; Kruskal–Wallis test with corrected Dunn’s post hoc test) differences and tendencies (● *p* < 0.1; Kruskal–Wallis test with corrected Dunn’s post hoc test) when compared to the untreated (CTRL, *n* = 7) group. The asterisk and dot in red correspond to significant differences between treatment groups (Ca^2+^ *n* = 7; µsCaEP *n* = 7; nsCaEP *n* = 8).

Two different duration calcium EP protocols (μsCaEP—1.5 kV/cm × 100 μs × 8, 1 Hz; nsCaEP—4 kV/cm × 700 ns × 200, 1 MHz) were applied on a Lewis lung carcinoma tumor model in vivo using parallel plate electrodes. Firstly, we assessed the treatment effect on C57BL/6J mice tumor growth changes (Figure 1A). We observed that calcium EP significantly attenuates tumor growth. Statistically significant tumor growth delay was observed in both calcium EP groups compared to calcium by itself. In the case of nsCaEP-treated mice, growth regression was even more apparent and resulted in statistically significant differences compared to untreated tumor-bearing mice (*p* < 0.05).

In Figure 1B individual responses of separate animals to the treatment are presented. It can be seen that all of the tumors responded to CaEP treatment; however, there is a variation in treatment efficacy. NsCaEP, on average, resulted in a better treatment for tumor growth inhibition. The variation within a group could be attributed to non-homogeneous electric field distribution within the tumor due to the application of non-invasive plate electrodes [48].

Moreover, for tumor induction, the luminescent LLC1-Luc cell line was used to visualize tumors in vivo. Mice tumor luminescence with an IVIS Spectrum device system was visualized before, immediately after (Day 0), and 10 days after the treatment (Day 10). The luminescence enabled better positioning of the electrodes and also served as a confirmation of the treatment efficacy on Day 10, while the necrotic scab was still present, which frequently makes volumetric measurement non-accurate. As can be seen in representative tumors luminescence images (Figure 1C), after calcium treatment, tumor luminescence was reduced several-fold. Thus, used calcium in combination with electroporation kills cancer cells shortly after the treatment, which is not the case for bleomycin-based therapies.

**Figure 2:**
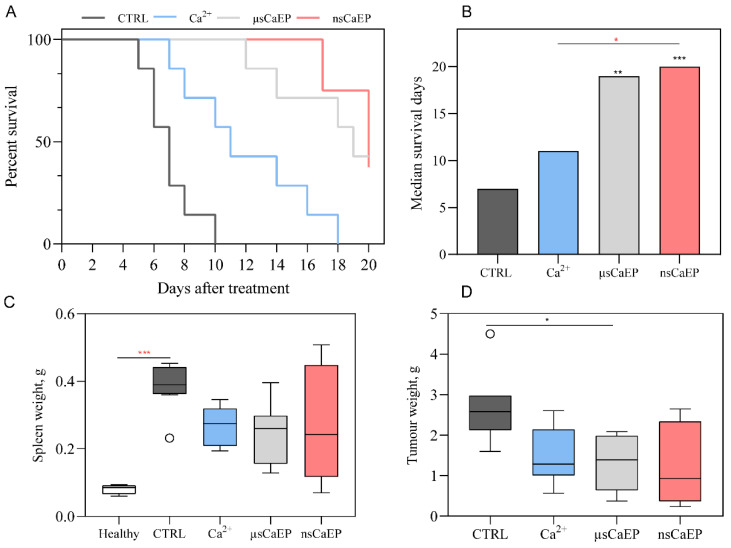
(**A**) Kaplan–Meier survival curves of mice with LLC1-Luc tumors. (**B**) Median survival days. The Mantel–Cox test was used for the statistical evaluation of mice survival. (**C**) Spleen and (**D**) tumor weight. CTRL—untreated tumor-bearing mice; Ca^2+^—tumor-bearing mice treated with CaCl_2_ (in 0.9% NaCl solution); μsCaEP—1.5 kV/cm × 100 μs × 8, 1 Hz; and nsCaEP—4 kV/cm × 700 ns × 200, 1 MHz protocol. The black asterisk (*) corresponds to statistically significant (* *p* < 0.05, ** *p* < 0.005; *** *p* < 0.0005) differences compared to the untreated (CTRL *n* = 7) group, where the red asterisk corresponds to significant differences between treatment groups (Ca^2+^ *n* = 7; µsCaEP *n* = 7; nsCaEP *n* = 8).

Further, untreated and differently treated groups of C57BL/6J mice were compared by survival rates (Figure 2A,B). Mice that received EP treatment exhibited significantly longer survival compared to untreated tumor-bearing mice (CRTL). Significant difference in median survival days between Ca^2+^ and nsCaEP-treated group was detected (*p* < 0.05). Untreated CTRL mice had a median survival of 8 days, while treated mice had a median survival that ranged 1.5–2.8 times longer than CTRL. Specifically, Ca^2+^ had a median survival of 11 days, μsCaEP had a median survival of 19 days, and nsCaEP-treated mice had a median survival of 20 days. Fully recovered mice were observed only in calcium electroporation treatment groups (μsCaEP and nsCaEP).

Moreover, at the end of the experiment, spleen and tumor weight were evaluated in different groups (Figure 2C,D). We have observed an increased spleen weight in all groups compared to healthy mice spleens. Moreover, the spleen weight of untreated tumor-bearing mice was, on average, higher compared to treatment groups, indicating that tumor-bearing mice had enlarged spleens.

To assess the association of immune response and spleen weight in mice, we have investigated the immune cell subsets in different organs.

### 2.2. Immune Cell Subsets in Spleen, Lymph Nodes, Blood, and Tumors

First, the T lymphocyte changes in tumors, lymph nodes (LN), spleens, and blood were assessed.

**Figure 3:**
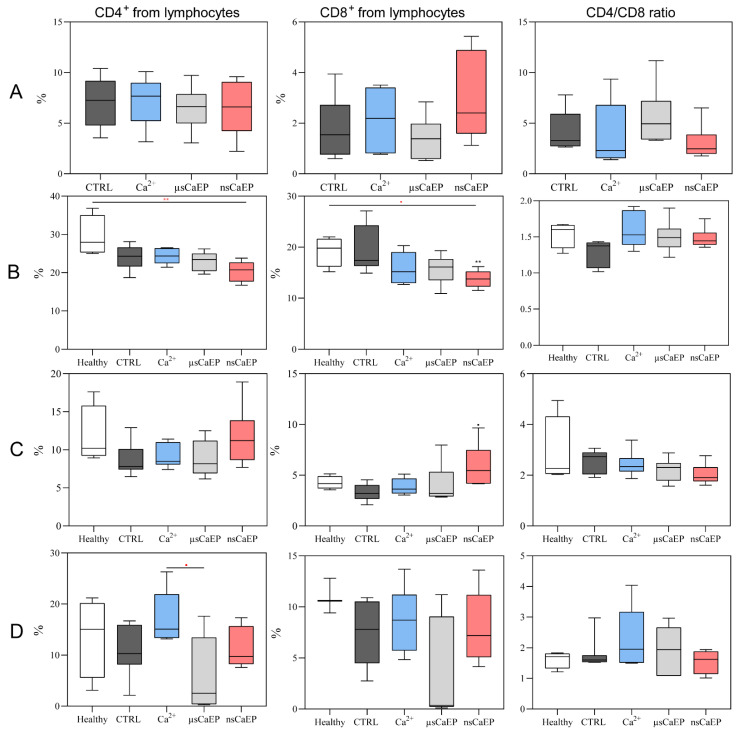
T lymphocyte populations in mice tumors (**A**) and organs, where lymph nodes are presented in (**B**), spleens in (**C**), and om blood (**D**). Cytometry was performed using a BD FACSAria III flow cytometer. Symbols in black corresponds to statistically significant (** *p* < 0.005) differences and tendencies (● *p* < 0.1;) compared to the untreated (CTRL *n* = 7) group, where the asterisk and dot in red correspond to significant differences between treatment groups (Ca^2+^ *n* = 7; µsCaEP *n* = 7; nsCaEP *n* = 8).

As reported in Figure 3, CD4/CD8 T cells ratio did not significantly change after EP treatment. However, a significant decrease in the percentage of helper (Th; CD4^+^) and cytotoxic T cells was noticed in the lymph nodes of nsCaEP-treated compared to untreated tumor-bearing (CTRL) mice. At the same time, a statistically significant increase in the percentage of cytotoxic T cells in spleen (compared to CTRL) was detected.

The percentages of blood circulating T cells were similar in all groups, except there was a difference in CD4^+^ + T calls between μsCaEP and calcium only treated groups.

Further, we defined different populations of cytotoxic and helper T cells by analyzing CD44 and CD62L surface markers. The expression pattern of these markers is contingent upon the functional state of T lymphocytes. During the primary immune response, activated T cell clones lose CD62L expression and acquire CD44 expression (CCD62L^−^CD44^+^), while central memory T cells express both surface markers (CD44^+^CD62L^+^) [49]. Here, we observed that effector and central memory T cells population changes after calcium electroporation treatment in tumors, lymph nodes, spleens, and blood circulation.

**Figure 4:**
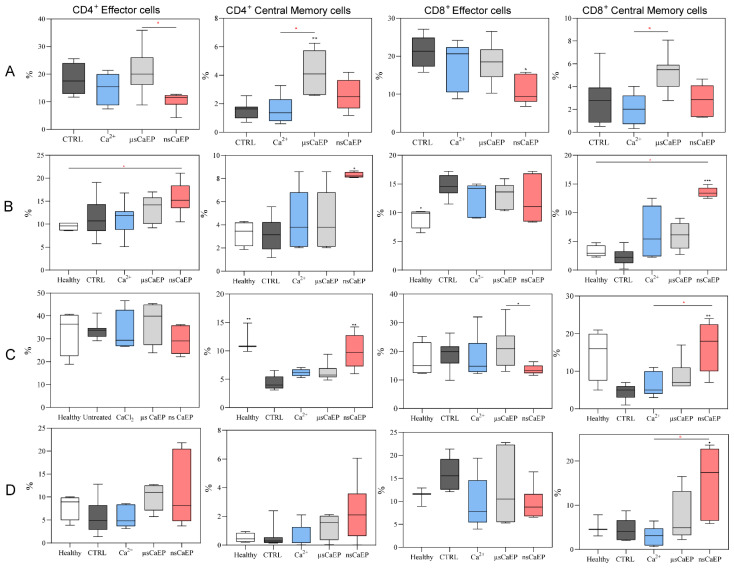
CD4^+^ and CD8^+^ T lymphocytes effector (CD62L^−^CD44^+^) and central memory (CD62L^+^CD44^+^) populations in mice tumors (**A**), lymph nodes (**B**), spleens (**C**), and blood (**D**). Cytometry was performed using a BD FACSAria III flow cytometer. Symbols in black corresponds to statistically significant (* *p* < 0.05, ** *p* < 0.005, *** *p* < 0.0005) differences and tendencies (● *p* < 0.1;) compared to the untreated (CTRL *n* = 7) group, where the asterisk and dot in red correspond to significant differences between treatment groups (Ca^2+^ *n* = 7; µsCaEP *n* = 7; nsCaEP *n* = 8).

As can be seen in Figure 4, a significant decrease in CD8^+^ T cells was observed in the nsCaEP-treated group when compared to untreated or tumor-bearing mice. At the same time, the percentage of Th central memory cells was significantly increased only in μsCaEP-treated mice tumors compared to untreated mice.

A significant increase in central memory CD4^+^ and CD8^+^ T cells percentage was observed in nsCaEP-treated mice lymph nodes when compared to CTRL (Figure 4B). During analysis of splenic central memory CD4^+^ and CD8^+^ T, we could see that the percentages of these splenic cells after nsCaEP treatment were similar to healthy mice. The percentage of blood circulating effector and central memory helper T cells did not differ in any of the mice groups. Remarkably, a significant increase in blood circulating cytotoxic central memory T cells was detected after calcium-mediated nsEP treatment, which was not the case for μsCaEP (Figure 4D).

**Figure 5:**
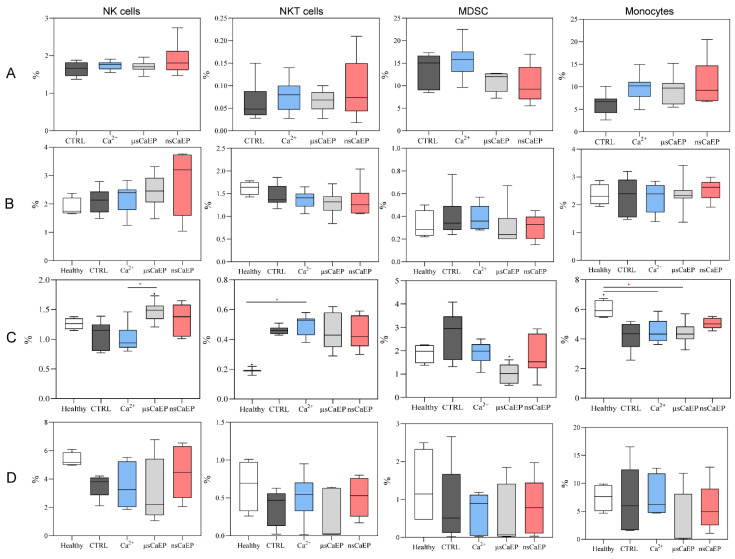
NK, NKT, MDSC and monocytes populations mice tumors (**A**), lymph nodes (**B**), spleens (**C**) and blood (**D**). Cytometry was performed using a BD FACSAria III flow cytometer. Symbols in black corresponds to statistically significant (* *p* < 0.05;) differences and tendencies (● *p* < 0.1;) compared to the untreated (CTRL *n* = 7) group, where the asterisk and dot in red correspond to significant differences between treatment groups (Ca^2+^ *n* = 7; µsCaEP *n* = 7; nsCaEP *n* = 8).

We have also investigated how the treatment influences percentages of NK (CD3^−^CD49b^+^), NKT (CD3^+^CD49b^+^) cells, myeloid-derived suppressor cells (MDSC; Gr1^+^CD11b^+^), and monocytes (Gr1^−^CD11b^+^) in mice tumors, lymph nodes, spleens, and blood. The results are summarized in Figure 5.

There were no changes in the percentage of tumor-associated NK and NKT cells between the groups (Figure 5A). Upon further analysis of the lymph nodes, none of the examined immune cell populations showed any differences between the groups of mice (Figure 5B). The percentage of splenic monocytes decreased in μsCaEP-treated mice compared to the healthy group (Figure 5C). According to the results shown in Figure 5D, there were no significant differences in the number of blood circulating NK, NKT, MDSC, and monocytes between the groups. For further analysis, blood-circulating antitumor IgG antibodies were assessed.

### 2.3. Calcium Electroporation Stimulates Humoral Immune Response

**Figure 6:**
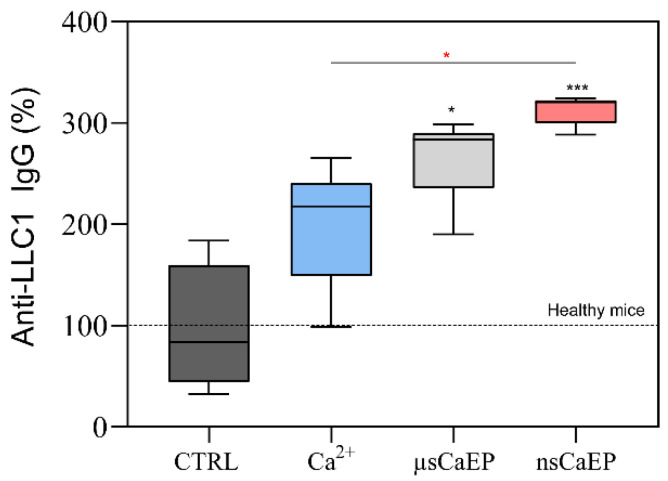
The relative percent of anti-LLC1 IgG antibodies. Data were normalized according to tumor-free, healthy mice. Flow cytometry was performed using Amnis FlowSight. The black asterisk (*) corresponds to statistically significant (* *p* < 0.05, *** *p* < 0.0005;) differences compared to the untreated (CTRL n = 7) group, where the red asterisk corresponds to significant differences between treatment groups (Ca^2+^ n = 7; µsCaEP n = 7; nsCaEP n = 8).

The relative percentage of anti-LLC1 IgG antibodies against surface and intracellular antigens was assessed (Figure 6).

Different modalities of calcium electroporation resulted in a significant increase in anti-LLC1 IgG antibodies compared to healthy or untreated tumor-bearing mice (CTRL). Treated mice had 2–3.2 fold higher levels of antitumor antibodies than CTRL mice. Notably, after nsCaEP treatment, the levels of antitumor antibodies were the highest.

## 3. Discussion

In previous studies, we observed that doxorubicin nano-electrochemotherapy could be used to eliminate SP2/0 tumors in BALB/c mice [24]. Additionally, we successfully showed that sub-microsecond range irreversible CaEP can be used for the eradication of myeloma tumors in vivo and that the treatment triggers an immune response [26]. Nevertheless, the application of nanosecond protocols is inferior to microsecond ones for drug delivery (e.g., bleomycin) [20] if reversible electroporation is used and the pulse repetition frequency is low [21]. In order to compensate for the lack of significant electrophoretic components and increase the stability and size of the pores, a new modality of high-frequency electroporation (MHz range) can be used [45,46]. Previously, we have shown that high-frequency nsECT with bleomycin can be as good or superior to ESOPE protocols [27]. However, calcium electroporation is gaining more and more popularity due to accessibility and the capabilities to modulate a systemic immune response. Therefore, in this study, we have used the MHz range high-frequency nsEP for calcium-mediated ECT, which was not performed before. For better consolidation of knowledge, we have compared the treatment with the ESOPE protocol and characterized the immune response in vivo.

In this work, it was shown that CaEP protocols *(*μsCaEP—1.5 kV/cm × 100 μs × 8, 1 Hz or nsCaEP—4 kV/cm × 700 ns × 200, 1 MHz) can be effectively used for tumor eradication in vivo. The tumor luminescent visualization confirms the obtained tumor volumetric measurement results. Our results on ESOPE pulses are in agreement with CaEP effects reported in other studies [5,12,26,35,43,44]. More importantly, we have presented novel data on MHz nanosecond pulses, which can be used as a better alternative for ECT. High-frequency nanosecond pulses are advantageous due to the higher frequency component, which results in a more homogenous treatment (impedance mitigation) [50]. Moreover, due to the short duration, the amplitude of PEF can be significantly higher, which ensures an above-threshold electric field even in the parts of the tumor that are not in direct contact with the electrode [19]. As a result, nsPEF protocols can trigger reversible electroporation and ESOPE-equivalent drug delivery even with pulses that vary in PEF amplitude by more than 30% [21] and compensate PEF non-homogeneity in heterogeneous tissue. The acquired experimental data confirms this hypothesis. Additionally, we could observe that the nsPEF triggered reduced muscle contractions when compared to μsCaEP, which is an expected result. Application of bipolar pulses should be even more effective in terms of impedance mitigation and reduction of muscle contractions [50]; however, the cancellation phenomenon will take place [51]. Therefore, considering available knowledge and our data, we speculate that application of even higher frequency (2–10 MHz) compressed monopolar pulse bursts should be a priority for transitioning ECT to the nano-range.

Moreover, we have determined an increased spleen weight in tumor-bearing untread mice. In the case of effective treatment, the spleen weight and its size were decreased and were comparable to healthy mice in the case of fully recovered animals. It is reported that the spleen plays a significant role in extramedullary hematopoiesis and tumor immunotolerance, and changes in spleen weight have been observed during tumor progression [52], which is in agreement with our study.

Additionally, we have determined the changes in splenic, tumor-associated, and draining lymph nodes, and blood-circulating immune cell subpopulations in CaEP-treated and untreated mice. First, the CD4^+^ and CD8^+^ T cells were analyzed since they are the primary lymphocyte populations involved in cell-mediated immunity, playing a pivotal role in facilitating robust immune responses against tumors. We speculate that differences in the tumor-associated CD8^+^ T cells after nsCaEP treatment in spleens and lymph nodes could be associated with cell-mediated immunity promotion. These findings agree with the pancreatic cancer irreversible electroporation treatment study [53]. Moreover, earlier studies have demonstrated robust immune protection achieved through nsEP tumor ablation in mouse breast [54] and rat hepatocellular cancers [55].

We have also analyzed effector and central memory populations of CD4^+^ and CD8^+^ T lymphocytes and a significant decrease in effector tumor-associated Tc lymphocytes was observed after nsCaEP treatment when compared to CTRL mice. Moreover, compared to untreated control mice, CD4^+^ and CD8^+^ central memory T cells in the spleen and lymph nodes significantly increased after nsCaEP treatment, which was not in the case for μsCaEP treatment. Our findings suggest that the use of nanosecond pulses could have some effect on long-term immune memory formation. Similar induction of splenic memory T cell response following nsEP treatment was reported by Guo et al. [54]. Moreover, comparable tendencies of effector and memory T cells dynamics after the treatment were reported in our study with irreversible calcium electroporation [26] and in He et al.’s research on irreversible electroporation with pancreatic cancer [56]. An increase in central memory T cells can be interpreted as a good sign due to central memory T cells’ ability to exhibit elevated cytokine production, enhanced cytotoxic activity in vitro, and the potential to demonstrate superior efficacy in eliminating established tumors in mice. Memory T lymphocytes can be characterized by an enhanced ability to migrate into the lymph nodes where antigen-presenting dendritic cells (DCs) can present antigens to them in the context of major histocompatibility complex (MHC) molecules [57].

Additionally, an immune response suppressing MDSC cells was evaluated after CaEP treatment. A significant decrease in splenic MDSC was noticed after μsCaEP treatment compared to CTRL, while after nsCaEP treatment decrease was not observed. Our results agree with the literature-reported data that IRE, ECT, and CaEP treatment decrease the MDSC [19,44,54]. Moreover, an increased percentage of splenic NK cells after μsCaEP treatment might be related to diminished tumor growth and better survival rates, which is in agreement with Lisec et al. study [35].

At the same time, monocytes serve as a crucial link between innate and adaptive immune responses and can influence the tumor microenvironment through diverse mechanisms. These mechanisms include the induction of antitumor effectors and the activation of antigen-presenting cells, ultimately contributing to an anti-tumor immune response [58]. However, upon closer examination of the lymph nodes and blood-circulating immune cells, no discernible differences were observed in any of the analyzed populations among the various groups of mice.

Nevertheless, we have observed the emergence of tumor-specific antibodies in the mouse plasma. It is recognized that cancer patients often exhibit auto-antibodies against tumor-associated antigens [41]. Similarly, we obtained cancer auto-antibodies targeting surface and intracellular antigens of LLC1 tumor cells in mice. The levels of these antibodies were notably elevated in mice treated with CaEP, particularly in nsCaEP-treated mice blood plasma. The presence of IgG class-specific antibodies suggests the involvement of CD4^+^ T lymphocytes in the antitumor immune response.

## 4. Materials and Methods

### 4.1. Electroporation Setup and Parameters

A square-wave pulse generator (Vilnius, Lithuania) [59] was used in the study. The pulses were delivered using parallel plate stainless steel electrodes by compressing the tumor between the flat electrodes (2–4 mm gap). In order to ensure good electrical contact, the electrodes were lubricated with EEG and ECG Transound gel (EF Medica Srl, Caldaro, Italy). The voltage has been adjusted to induce 1.5 kV/cm (100 µs × 8, 1 Hz, ESOPE) or 4 kV/cm (700 ns × 200, 1 MHz) electric field. The ESOPE protocol served as a reference for comparison of the treatment efficacy induced by high-frequency nanosecond pulses. The summary of electroporation protocols is shown in Table 1.

**Figure 7:**
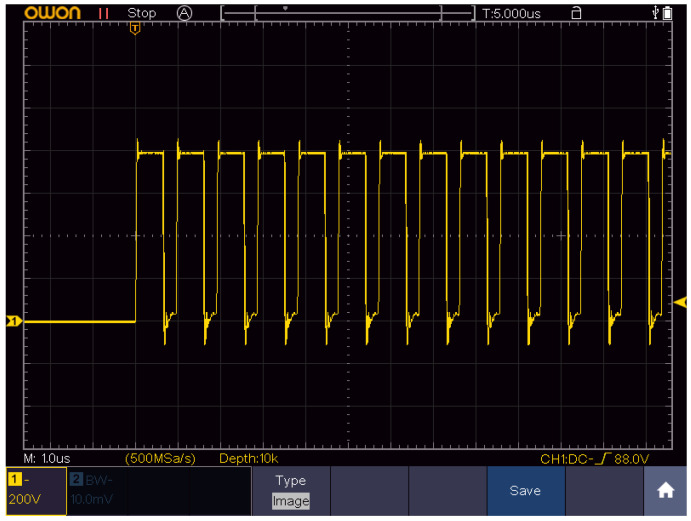
The measured waveform of the 700 ns square-wave pulses delivered at 1 MHz repetition frequency acquired using TAO3000 oscilloscope (Owon Technology, Richmond Hill, ON, Canada).

The energy of the burst was estimated by multiplying the amplitudes of the current and voltage measurements, pulse duration, and the number of pulses. It should be noted that only one instance in each group was evaluated and the effects of the electrode gap on the pulsed current were not considered. It can be seen that for both protocols, the energy of the whole burst is less than 0.5 J; thus, the effects of Joule heating are negligible. The pulse waveform of the nanosecond burst is shown in Figure 7.

### 4.2. Cells

Lewis lung carcinoma (LLC1) cell line was obtained from the National Cancer Institute (Vilnius, Lithuania), and luciferase-expressing (LLC1-Luc) cell line was developed in State Research Institute Centre for Innovative Medicine (Vilnius, Lithuania) [19]. Cells were maintained in RPMI 1640 medium supplemented with 10% fetal bovine serum (FBS), 2 mM glutamine, 100 mg/mL streptomycin, and 100 U/mL penicillin (reagents were obtained from Gibco, Thermo Fisher Scientific, Waltham, MA, USA).

### 4.3. Animals and Tumor Model

Female C57BL/6J 6–8-week-old mice were bred and housed in the mouse facility of the State Research Institute Centre for Innovative Medicine (Vilnius, Lithuania). A total of 1 × 10^6^ LLC1-Luc cells were injected subcutaneously (s.c.) on the left flank to establish carcinoma tumors. Moreover, healthy, tumor-free, age-matched mice were included in this study as a healthy control group.

**Figure 8:**
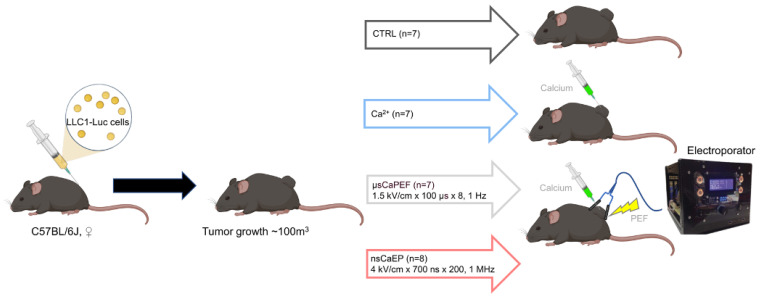
Schematic experimental design.

After 1–2 weeks, established subcutaneous tumors reached 100 mm^3^ (Day 0) mice were randomized into 4 groups, and treatment was applied (Figure 8). Before the treatment, mice’s backs were shaved and depilated using 8% Na_2_S aqueous solution and then rinsed with water. Then, before the electroporation, a single injection of 250 mM calcium chloride (CaCl_2_) in 0.9% NaCl solution was delivered into the tumors (approximately half of the tumors volume). After 5 min, mice were treated with EP, and the procedure was performed under anesthesia (3% isoflurane and oxygen gas mixture). The general scheme of the experiment is shown in Figure 8.

Mice tumor volumetric measurements and health status were observed every 2 to 3 days after the treatment. The volumes of tumors (mm^3^) were calculated using the formula:

V = (Length × Width^2^ × π)/6, where π = 3.1416.

The luminescence of tumors was assessed using IVIS Spectrum and Living Image Software (Caliper/Perkin Elmer, Akron, OH, USA). Prior to the treatment, mice received 30 mg/mL D-luciferin (Promega, Madison, WI, USA) solution (150 µL/mouse) via intraperitoneal injection. Luciferase intensity was visualized before the treatment, right after the treatment, and 10 days after the treatment. Luminescence was expressed as the photons/sec/region of interest (ROI) by subtracting the background luminescence of the same size region.

The consent to perform animal experiments was obtained from the State Food and Veterinary Service (approval no. G2-145), carrying out the study strictly according to the Guide for the Care and Use of Laboratory Animals.

### 4.4. Tissue Preparation

The blood was collected from the hearts of sacrificed mice at the end of the experiment in the tubes containing ethylenediaminetetraacetic acid (EDTA) anticoagulant in order to obtain the plasma. The plasma was stored at −20 degrees for the antitumor antibodies’ determination, and the cells were stained for analysis with flow cytometry.

Tissues’ single-cell suspension was prepared by mashing the spleen, lymph nodes, and tumors through a cell strainer (70 µm). Splenocytes were treated with 0.16 M NH_4_Cl ammonium chloride to lyse mouse erythrocytes. For tumor-infiltrating lymphocytes (TILs), the tumor tissues were enriched using Ficoll-Paque Premium (GE Healthcare, Chicago, IL, USA). All prepared single-cell suspensions were washed with phosphate buffer saline (PBS) and resuspended in a small amount of buffer for flow cytometry (FACS; 2% FBS and 0.1% NaN_3_ in PBS).

### 4.5. Flow Cytometry

Cell populations were identified by using different sets of fluorochrome-labeled monoclonal antibodies (mAbs): anti-CD4 PerCP Vio770, anti-CD44 FITC, anti-CD8a Alexa Fluor700, anti-CD25 PE, anti-CD127 APC, anti-CD62L PE Cy7, anti-FR4 (BV421), anti-CD3 FITC, anti-Gr-1 PE, anti-Dx5 APC Cy7, anti-CD11b PE-Texas Red. For the dead cell discrimination LIVE/DEAD near-IR (ex. 633 nm) and violet (ex. 405 nm) viability dyes were used (Thermo Fisher Scientific, USA). The measurement of immune organs was performed with BD FACS Aria III flow cytometer (BD Biosciences, San Jose, CA, USA), and data analysis was made with FlowJo 10.8.2 software (BD, Franklin Lakes, NJ, USA). The used antibodies are presented in the Supplementary Data (Table S1).

### 4.6. Antitumor Antibodies Determination

Mice blood plasma was collected at the end of the experiment and used to analyze anti-LLC1 antibodies against both intracellular and extracellular LLC1 cells’ antigens. Firstly, the LLC1 cells were fixed using paraformaldehyde (PFA, 2%) in PBS buffer for 10 min at 37 °C. After the incubation cells were washed and centrifuged at 300× *g* for 5 min at 4 °C and permeabilized using ice-cold Triton X-100 (0.2%) in PBS for 9 min on-ice. Afterward, cells were instantly resuspended in FACS buffer and centrifuged at 300× *g* for 5 min at 4 °C. Next, the cells were filtered through a strainer (70 µm) and diluted in Fc-Block to 0.3 × 10^6^ cells per sample. The cells were incubated for 1 h in diluted mice plasma in PBS buffer. Subsequently, the cells were washed with PBS solution, followed by centrifugation at 300× *g* for 5 min at a temperature of 4 °C. Then, samples were incubated with goat anti-mouse IgG AF488 antibodies (eBioscience, Invitrogen, Thermo Fisher Scientific, Waltham, MA, USA) for 30 min on ice, protected from light. As for negative control, cells were incubated only with goat anti-mouse IgG AF488 antibodies, without mice plasma. The Amnis FlowSight cytometer (Amnis Luminex/MilliporeSigma, Burlington, MA, USA) was used to measure relative fluorescence, and data analysis was performed using ImageStream IDEAS (Amnis Luminex/MilliporeSigma, Burlington, MA, USA).

### 4.7. Statistical Analysis

For non-normally distributed data, a non-parametric Kruskal–Wallis test with corrected Dunn’s post hoc test was performed to determine if the tumor volumes, organs weight, and cytometry results of different organs significantly differ among groups of treated and untreated mice. The *p* < 0.05 was considered significant and *p* < 0.1 tendentious for all statistical analyses. Log-rank (Cox–Mantel) test was used to analyze mice survival data (Kaplan–Meier survival analysis). Statistical analyses were performed with GraphPad Prism 8 software (San Jose, CA, USA).

## 5. Conclusions

To summarize, our results provide compelling preclinical evidence supporting the use of high-frequency (MHz range) nanosecond calcium electroporation as a promising approach for the effective treatment of cancer, which can serve as a viable alternative to ESOPE treatment. Moreover, we have shown that CaEP treatment induced a systemic antitumor immunity and long-term memory as well as a T cell-dependent antibody response. The capability to modulate the immune system with CaEP could be highlighted as a promising tool for controlling metastases and research into possible synergistic effects with immunotherapies.

## Figures and Tables

**Table 1:** Electroporation protocols employed in the study.

Protocol	Pulse Parameters	Current (A)	Energy (J)
nsCaEP	4 kV/cm × 700 ns × 200, 1 MHz	3.4	0.38
μsCaEP	1.5 kV/cm × 100 μs × 8, 1 Hz	1.2	0.29

## Data Availability

Data is contained within the article and supplementary material.

## Supplementary Materials

The following supporting information can be downloaded at: https://www.mdpi.com/article/10.3390/ph16081083/s1, Table S1: List of antibodies used.
