# Supplementary material for: Calcium Electrochemotherapy for Tumor Eradication and the Potential of High-Frequency Nanosecond Protocols
Source: Pharmaceuticals (Basel). 2023 Jul 31;16(8):1083. doi: 10.3390/ph16081083 (PMC10460074; doi:10.3390/ph16081083)
Supplement: Supplementary file 1 [file pharmaceuticals-16-01083-s001.zip › pharmaceuticals-2513389-supplementary/Supplementary.pdf]

Supplementary data

*Table S1. List of antibodies used.*

| Antibody | Resource | Identifier |
|---|---|---|
| PerCP-Vio700 Anti-CD4 | Miltenyi Biotec | 130-102-271 |
| FITC Anti-CD44 | BD | 553133 |
| AF700 Anti-CD8 | Invitrogen | 2075802 |
| APC Anti-CD127 | Invitrogen | 2480836 |
| BV421 Anti-FR4 | BD | 744119 |
| PE/Cy7 Anti-CD62L | Biolegend | 104417 |
| PE Anti-CD25 | MACS | 120-001-294 |
| FITC Anti-CD3 | BD | 53198 |
| AF488 Goat anti-mouse IgG | Invitrogen | 2090562 |
| APC Cy7 Anti-Dx5 | Invitrogen | A15420 |
| PE-TxRed Anti-CD11b | Invitrogen | 2159105 |
| PE Anti-Gr1 | MACS | 120-002-245 |
| LIVE/DEAD near-IR (ex. 633 nm) | Invitrogen | 2339909 |
| LIVE/DEAD violet (ex. 405 nm) | Invitrogen | 2328981 |
